# NARRATIVE-DRIVEN RECRUITMENT MESSAGING TO ENHANCE ETHNORACIAL DIVERSITY IN AD RESEARCH

**DOI:** 10.1093/geroni/igad104.3485

**Published:** 2023-12-21

**Authors:** Jennifer Lingler, Melissa Knox, Ishan Williams, Judy Cameron, Rena Robinson, Lisa Tamres, Melita Terry, Dianxu Ren

**Affiliations:** University of Pittsburgh School of Nursing, Pittsburgh, Pennsylvania, United States; University of Pittsburgh School of Nursing, Pittsburgh, Pennsylvania, United States; University of Virginia, Charlottesville, Virginia, United States; University of Pittsburgh School of Medicine, Pittsburgh, Pennsylvania, United States; Vanderbilt University, Nashville, Tennessee, United States; University of Pittsburgh School of Nursing, Pittsburgh, Pennsylvania, United States; University of Pittsburgh School of Medicine, Pittsburgh, Pennsylvania, United States; University of Pittsburgh School of Nursing, Pittsburgh, Pennsylvania, United States

## Abstract

Underrepresentation of persons of color in research on Alzheimer’s disease (AD) is a longstanding issue that is receiving unprecedented attention following reports of severely inadequate racial and ethnic diversity in clinical trials of the field’s first anti-amyloid therapies to secure Food and Drug Administration approval. The Recruitment Innovations for Diversity Enhancement (RIDE) Study is one of several federally supported initiatives to address key aspects of this complex and multi-faceted challenge facing the AD research community. RIDE is a multi-phase study to determine the effect of a culture-centric narrative campaign on recruitment of African American adults into clinical research on AD and related disorders. This presentation aims to contrast the RIDE study’s “narrative driven approach” to “traditional research messaging” with approaches typically found in research recruitment materials. Narrative driven messaging draws on the words, perspectives and stories from members of a culture-sharing group themselves, while traditional approaches aim to educate research candidates about potential benefits and risks of participation using language scripted from investigators’ perspectives. We will demonstrate how the narrative driven approach in RIDE focuses on a) prioritizing information that active research participants from a given culture-sharing group identify as most important and b) sharing that information in the form of culturally relevant stories. The potential benefits of this approach include a concrete, replicable strategy that can be readily implemented by other researchers. This is significant because recruiting representative samples in AD research is foundational to conducting the studies necessary to understand and combat race-based cognitive health disparities.

